# Modern Medico Psychology and Psychiatry

**Published:** 1895-08-17

**Authors:** T. S. Clouston

**Affiliations:** Lecturer on Mental Diseases, Edinburgh University, and Physician Superintendent Royal Asylum, Morningside


### Modern Medico-Psychology and Psychiatry.

By T. S. Clouston, M.D., Lecturer on Mental Diseases, Edinburgh University, and Physician Superintendent Royal Asylum, Morningside.

### CRIMINOLOGY.

The study of imperfect, arrested, and unrelational development would not be complete without some mention of the modern school of "criminology." The general conclusions of that school as represented by Lombroso, its most famous professor, are that criminality is largely due to weakness of brain, whether weakness of intellect, of natural feeling, of control, or of social instinct, and that environment, education, and example are less accountable for criminality on the whole than are heredity and arrestment or perversion in the development of the brain as regards its highest qualities. To prove this contention, criminals of all kinds, of all ages, and of all countries have been studied, described, assorted, and photographed. The greater the villain the better was the specimen; and the habits, the ways, the whole life history of him have been minutely investigated. His brain, his eyes, his ears, his face, his whole body in its every part, have been measured and described. Specimens of the class of both sexes, having been photographed and drawn, are depicted in endless rows in Lombroso's pages for comparison with each other, and with ordinary men and women. Lombroso's conclusions are definite and far-reaching, and if absolutely true, imply a recasting of our present system of trying and treating our criminals. For if men are "villains by necessity; fools by heavenly compulsion; knaves, thieves, and treacherous by spherical predominance; drunkards, liars, and adulterers by an enforced obedience of planetary influence," there is little use in trying to reform and make them honest citizens.

No one can study the inmates of a large prison without seeing that the majority of them are poor specimens of humanity in mind and body. They are distinguished by a general falling away from the type of ideal humanity in all directions. Their heads are to a large extent badly shaped, their facial appearance and expression are commonly poor, their hard palates are largely deformed, their movements are ungraceful, and there are frequent marks of neurotic and general degeneration. Yet all these facts do not prove a "criminal type," or even a series of different criminal types. Let any observer next go and examine with the same minutely observant eye the people of the class from whom most of the criminals come, but who have never been criminals at all. He will find the same general evidences of a low type of body and mind, but he will soon observe that in many of the actual criminals all the evidences of degeneracy are markedly accentuated. It seems to be true that the majority of criminals are just the worst and lowest of a degenerate stratum of society, most of whom, criminals and non-criminals, bear on their bodies and minds certain "stigmata" of degeneration.

Is the study of the mental and bodily characteristics of the criminal by the modern scientific methods therefore useless? By no means. It clearly demonstrates there are among criminals many whose brain power is so low that they are virtually criminals by organic necessity if subjected to certain temptations. One most striking fact is brought out in the "judicial statistics." It is that about half the criminals on their first conviction are under 25 years of age. Now this at once, to my mind, gives criminality a physiological and pathological aspect. It needs a certain power of mental inhibition to resist temptation, especially among the low, the poor, and the degenerate. That power is not completed, in a full physiological sense, till the full development of the brain at 25. This relationship of much of our criminality to adolescence is a strong argument for the exertions of the practical reformatory educators of young criminals. The fact of unfavourable heredity does not in any way militate against such a course. Physiologists know that the brain is then plastic, and that many hereditary weaknesses can then be antagonised. Let us, therefore, go on studying our criminals by all the methods of the criminologist, and let us also go on trying to reform them by every moral, religious, and educational means, especially when they are young; but let us reserve our final conclusions as to the respective strengths of heredity and environment till we get more facts. At present these facts indicate a degenerate bodily condition as associated with a large part of the criminality of the world, but not a distinct criminal type, and leave much room for the hypothesis that it is, after all, more a question of degree than of kind. Which of us possesses perfectly symmetrical bodies or perfectly "well-balanced" minds? Why are some of us entirely devoid of musical sense, some bereft of any arithmetical power, some born with "bad," uncontrollable tempers, some with very indifferent social faculties? Every one of these things shows unrelational development of brain in some degree, and there is no line of demarcation between the degrees that are consistent with law-abidingness, social order, and civilisation, and the degrees that, were they common, would render further evolution impossible and dissolve society in a generation. The children that suffer from nervous and other congenital or developmental defects and arrests should be nurtured, trained, and treated entirely on physiological lines. I believe they should have a diet mostly consisting of milk, farinaceous materials, fats, fruits, and vegetables. They should be brought up on routine and unexciting lines. Infinite pains should be taken to form good habits and when formed, they should never be let slip. Muscularly, organically, mentally, and morally, let a rigid system and order prevail. Their occupation should be mechanical rather than mental. Such cases occurring in a mild degree in the families of the rich suffer from the ambition of their parents. They are designed for work for which they are utterly unfitted. In our highly-organised society, especially in cities, they have a bad chance. The country is their natural habitat, and the more quiet country life they can have the better for them. For the proper education of some of this class of persons we also need special schools where they would really be detained under legal authority and subjected to a special training that might so far antagonise their weak points of development.

